# A qualitative evaluation of hospital versus community-based management of patients on injectable treatments for tuberculosis

**DOI:** 10.1186/s12889-018-6015-3

**Published:** 2018-09-17

**Authors:** D. B. Cohen, M. Phiri, H. Banda, S. B. Squire, I. Namakhoma, N. Desmond

**Affiliations:** 1 Malawi Liverpool Wellcome Clinical Research Programme, Box 30096, Blantyre 3, Malawi; 2 Liverpool School of Tropical Medicine, Pembrooke Place, Liverpool, L3 5PH UK; 3 University of Sheffield, Medical School, Beech Hill Road, Sheffield, S10 2RX UK; 4 REACH Trust, Box 1597, Lilongwe, Malawi

**Keywords:** Malawi, Tuberculosis, Community-based care, Injectors, Streptomycin, In-patient, OPAT

## Abstract

**Background:**

Patients being treated for recurrent or multidrug-resistant tuberculosis (TB) require long courses of injectable anti-tuberculous agents. In order to maintain strong TB control programmes, it is vital that the experiences of people who receive long-term injectables for TB are well understood. To investigate the feasibility of a novel model of care delivery, a clinical trial (The TB-RROC Study) was conducted at two central hospitals in Malawi. Hospital-based care was compared to a community-based approach for patients on TB retreatment in which ‘guardians’ (patient-nominated lay people) were trained to deliver injections to patients at home. This study is the qualitative evaluation of the TB-RROC trial. It examines the experiences of people receiving injectables as part of TB treatment delivered in hospital and community-based settings.

**Methods:**

A qualitative evaluation of the TB-RROC intervention was conducted using phenomenographic methods. Trial participants were purposively sampled, and in-depth interviews were conducted with patients and guardians in both arms of the trial. Key informant interviews and observations in the wards and community were performed. Thematic content analysis was used to derive analytical themes.

**Results:**

Fourteen patients, 12 guardians and 9 key informants were interviewed. Three key themes relating to TB retreatment emerged: medical experiences (including symptoms, treatment, and HIV); the effects of the physical environment (conditions on the ward, disruption to daily routines and livelihoods); and trust (in other people, the community and in the health system). Experiences were affected by the nature of a person’s prior role in their community and resulted in a range of emotional responses. Patients and guardians in the community benefited from better environment, social interactions and financial stability. Concerns were expressed about the potential for patients’ health or relationships to be adversely affected in the community. These potential concerns were rarely realised.

**Conclusions:**

Guardian administered intramuscular injections were safe and well received. Community-based care offered many advantages over hospital-based care for patients receiving long-term injectable treatment for TB and their families.

**Electronic supplementary material:**

The online version of this article (10.1186/s12889-018-6015-3) contains supplementary material, which is available to authorized users.

## Background

Each year, approximately 700,000 people globally are treated for recurrent tuberculosis (TB) and a further 125,000 are treated for multi-drug resistant tuberculosis [1]. People presenting with recurrent or multidrug-resistant disease require extended courses of treatment which include daily injections of anti-tuberculous medications, often for many months [2]. If the treatment of recurrent and drug resistant TB is to be successful, it is essential that models for the delivery of long-term injectables are feasible and affordable for health systems; but crucially that they are also acceptable to health system users.

There are various options for the delivery of long-term daily injectables as part of TB treatment, but each has major disadvantages. Prolonged admission to hospital is associated with hospital-acquired infections [3]; social and economic consequences for patients and their households [4]; and a disproportionate burden on health systems [5]. A number of community-based approaches to care have been implemented, but once again these often face barriers to success [6]. A system which involves healthcare workers travelling daily to patients at home in order to administer injections incurs additional expense and healthcare worker hours. The alternative is for patients to travel daily to a health facility, but this model simply transfers the time and financial burdens onto already vulnerable health system users.

The TB-RROC (TB Retreatment Regimen Outcomes and Care) Trial (ISRCTN05815615) aimed to assess a novel method of community-based management for patients with recurrent TB. These patients are prescribed a number of oral anti-tuberculous drugs plus the injectable drug *streptomycin* daily during the first two months of TB treatment [2]. Participants in the TB-RROC Trial received hospital or community-based care during the two-month period that they were receiving streptomycin injections. Community-based care involved training patient-nominated lay people (‘guardians’) to administer streptomycin injections to patients in their own homes. In many Sub-Saharan African countries, if a patient is admitted to hospital they are accompanied by a ‘guardian’ – a lay person, usually a family member or friend, who looks after them and provides much of their basic care including washing, dressing, feeding and even giving oral medications [7]. In the TB-RROC trial, adult patients were recruited after admission to the TB ward for treatment of recurrent TB. Two hundred and four participants were individually randomised to receive hospital or community-based care after the patient was medically fit for discharge and their self-appointed guardian had successfully been trained to deliver injections.

The aim of this qualitative study was to evaluate the experiences of the participants in the TB-RROC trial. This is the first study which specifically addresses user experiences of different models of care for patients who require long-term injections as part of TB treatment.

## Methods

### Theoretical framework

As the aim of the study was to describe the lived experiences of patients and guardians during TB treatment, it took a phenomenographic approach [8]. As such, it attempted to understand the variety of ways in which people experience and understand the phenomenon of being on TB treatment. An initial conceptual framework was developed based on review of the literature, discussion with key stakeholders, and the researchers’ own experiences of working within TB services in Malawi (Appendix 1).

### Data collection

The primary data were in-depth interviews with trial participants. Triangulation of the primary data was achieved by conducting a number of key informant interviews as well as observations of patients at home and in the ward. All participants in the TB-RROC trial and this qualitative evaluation were adults over the age of 16 years. Interviews were conducted in the local language (Chichewa), and recorded using a digital dictaphone. Pre-prepared topic guides were used for all interviews and field guides for all observations (see Additional file 1). After each data collection episode, a debrief session was held, and the guides were adapted iteratively throughout the study. Data were collected until it was felt that saturation had been reached and no new concepts were arising.

#### In-depth interviews with trial participants

Purposive sampling of consecutive TB-RROC trial participants was employed to ensure a broad range of perspectives were covered (Appendix 2). Depending on the sampling framework and willingness to participate, both members of a patient-guardian pair or only one member of the pair were recruited. Patients were approached as they completed their two-month course of streptomycin and interviewed within the first two weeks after finishing injections. This time point was chosen because participants still on intensive phase treatment under the care of the study team may not yet have felt able to talk freely and reflectively about the experience; but it was a time point not too far from the event which should have meant that participants were still able to recall clearly their experiences. Participants were interviewed individually either at the hospital or their own homes according to preference.

#### Key informant interviews

Key informants included a variety of health care workers (nurses, TB officers); other members of the community; and potential recruits who declined to participate in the trial.

#### Observations

The purpose of home visits within the TB-RROC trial was for the trial fieldworker to assess injections being given and to monitor participants progress on treatment. Qualitative observations were performed in the community by accompanying the trial fieldworker on randomly selected home visits. In order to minimise disruption to the process of the home visits, the qualitative researcher was introduced as a colleague of the field worker. They observed the home environment; the interaction between patient and guardian; interactions between study participants and other people in the household; interaction between study participants and fieldworker; and when possible the administration of injections. For observations on the ward, the researcher placed themselves in an unobtrusive place on the ward in order to observe the environment; clinical and non-clinical activities; and interactions between patients, guardians and healthcare workers. Patients on the ward were not formally introduced to the researcher, as this would have biased results. Observations in hospital only took place in public places, and therefore did not result in additional breaches of privacy or confidentiality.

#### Participant feedback

Two participant feedback sessions were held after key descriptive themes had been identified from the data. One session involved patients and guardians in the trial and the other involved healthcare workers. These sessions provided a means of validating the initial data analysis; and created an additional forum for participants to voice opinions and ideas.

### Data management and analysis

All interviews and group discussions were transcribed verbatim in Chichewa and then translated directly into English. For quality control purposes, a second transcriber reviewed a random 20% sample of all transcripts. Anonymised transcripts were imported into Nvivo10 QSR software for organising, management and analysis of data. Transcripts were inductively coded and analysed using Thematic Content Analysis. The units of meaning for analysis were text in English (words or phrases). Two coders (DC and MP) coded the first 5 transcripts and independently generated a coding structure, which were discussed in order to produce a unified coding schedule. This coding schedule was used to code further transcripts, and was adapted iteratively as new data were collected. Analytical themes were then generated by discussion between all six members of the research team.

## Results

In total, 26 in-depth interviews with TB-RROC trial participants and 9 key informant interviews were performed (Table 1). Six observations were performed – two in the community and four in the hospital. Four in-depth interviews and one key informant interview were performed with trial participants in Lilongwe, the remainder were performed in Blantyre.

**Table 1:** Characteristics of study participants

	Participant type	Gender	Care delivery method/Role
In-depth interviews (26)	Patients (12)	Male (7)	Community (5)
			Hospital (2)
		Female (5)	Community (2)
			Hospital (3)
	Guardians (14)	Male (7)	Community (6)
			Hospital (1)
		Female (7)	Community (2)
			Hospital (5)
Key informant interviews (9)	Healthcare workers (4)	Male (2)	TB Officers (2)
		Female (2)	Nurses on TB ward (2)
	Potential trial recruits who declined to participate (3)	Male (2)	Patients (2)
		Female (1)	Guardians (1)
	Other (2)	Female (2)	Other household members of trial participants

**Fig. 1:**
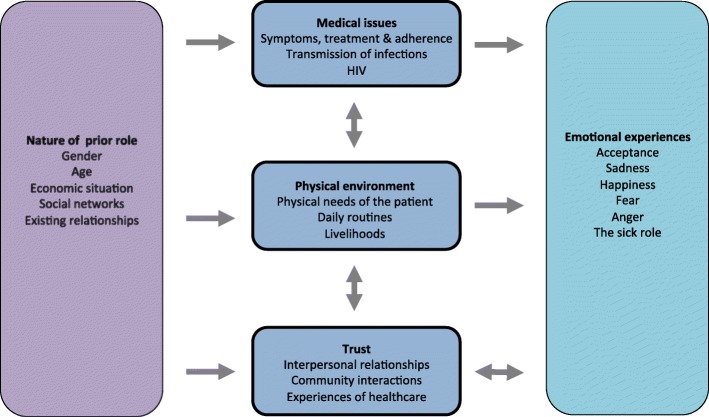
Experiences of patients and guardians during TB treatment

Experiences of TB treatment within the TB-RROC trial revolved around three main themes: medical issues, the physical environment, and trust. Each of these experiences were influenced by the nature of participants’ prior role in their community, and in turn resulted in a broad range of emotional experiences as shown in Fig. 1.

### Medical issues

#### Symptoms, treatment and adherence

Recurrent TB was the experience which linked every patient in the study. Recognition of the symptoms of TB was often enhanced by previous experiences of having had TB. Many patients felt that TB tablets were ‘too strong’, or ‘very powerful’ and some were concerned by the potential for more serious side effects during retreatment because of the longer duration and the addition of injections compared to their previous treatment.

Both patients and guardians managed in the community were aware of the potential complications of injections such as swelling, infection, or paralysis of the leg. Although expressed concerns about guardians doing injections were extremely common, the only description of an adverse clinical event was unrelated to the guardian having given injections (the patient developed a separate intercurrent illness and required re-admission to hospital). Nevertheless, both trial participants and healthcare workers recognised the importance of guardians receiving appropriate training before considering community-based management, and that only those patients who were well enough should be discharged:

*“The strategy should be executed depending on the condition of the patient ... people like these critically ill ones require being in the hospital for frequent monitoring by the doctors. But someone who does everything by herself, is strong... could be discharged to go and be injected at home if her guardian manages to inject so that she can perform other household chores.”*

*P9, Female patient, community-based care*

Concerns were expressed by trial participants as well as by nurses and TB officers about the *potential* for non-adherence to injections in the community. In particular it was perceived that guardians in certain positions – such as wives, or younger siblings – would be unable to insist on giving an injection to a patient who was refusing; or that arguments within families would mean that guardians would refuse to care for the patient properly. However, these remained perceived problems, and no patients or guardians described having missed an injection. In one situation, a guardian threatened to stop giving injections because of an argument over money with another family member which had nothing to do with the patient receiving injections, but after discussion within the family he did administer all doses. This demonstrated the potential for the injector role to be used as a control mechanism, however the conflict was rooted in existing relationships within the family rather than developing after the guardian took on the responsibility for injecting.

#### Transmission of infections

There was anxiety about guardians contracting infections (both TB and other infections) whilst caring for patients in the hospital. This issue was raised not only by guardians themselves, but also by nurses on the TB ward. These ‘medical issues’ were closely related to the ‘physical environment,’ and reducing transmission of infection was seen as a major advantage of community-based care, where the risk of contracting illness was perceived as lower:

*“The guardians are at risk of this same TB. I have seen a couple of women who came back to the hospital infected with TB two or three months after being guardians [in hospital] for their husbands.”*

*P34, Female nurse*

*“I also felt at risk of other infections and was worried … You would have a critically ill patient struggling to get to the toilet and soiling himself on the bed just next to you. I felt I was in danger of other infections than the one I was in the hospital for.”*

*P22, Male patient, hospital-based care*

Initial concerns were expressed by guardians about the risk of acquiring HIV from needle stick injury whilst doing injections, but they were more confident having received training. Some patients and guardians worried about having needles at home if there were children in the house, although by safely storing equipment, they all managed to avert their fears and none of the participants reported a needle stick injury. Although emotional responses of guardians to the possibility of contracting infections either during a hospital stay or in the community were negative (‘fear’ and ‘sadness’) the risk was always accepted.

### The physical environment

#### The physical needs of the patient

Both during admission and after discharge from hospital, care for patients was usually the task of one main guardian, but responsibility was often shared amongst family members and the community. The division of responsibility was determined largely by prior circumstances, such as existing economic situation, relationships and social networks.

#### Daily routines

Trial participants (both patients and guardians) found the ward environment distressing, and described in detail the unpleasant reality of daily life in hospital. The predominant feature was overcrowding, which affected people on many levels – the physical discomfort of not having enough space, the lack of privacy and dignity, difficulty in getting care, and lack of hygiene:

*“This time their nostrils have detected a bad smell. As they enquire from one another where the smell is coming from, they discover it is from the patient just next to them, the one with a small baby. She has been soiling herself up since morning and can’t bathe because there is no water.*

*‘The smell is too bad,’ one of them complains.*

*The woman doesn’t respond. She quietly walks to the sink just close to where I am to see if there is water, but still there is no water.* *‘So what do you do when it is like this?’* *I ask her*

*‘Nothing, we just wait until the water comes back,’ she replies”*

Field note, hospital observation 2

Admission to hospital caused massive, often disastrous, disruption to peoples’ usual daily routines. Although spending long periods on the ward affected guardians’ ability to work, the consequences for employment were generally profounder for patients than guardians. The impact of admission was more significant for those who previously had provider or carer roles within their household. However, there was no clear evidence that gender played a role in this effect, as a number of men had carer roles and many women worked.

Being able to continue normal activities such as going to school and church was a major advantage for those who were discharged after being randomised to receive community-based care. Being able to function more ‘normally’ at home often benefited patients emotionally. Many described feeling less like a patient once they went home, which was seen to aid recovery, and change their ‘sick role’ in the community.

*“When you are taking medication at home it feels like you are not on medication. It feels like you are just staying. When someone sees you, they say ‘Ah, he is not sick,’ when you are actually sick, because you are able to perform household work on your own. When you are in the hospital … all you think about its ‘I am in the hospital’ and the sickness worsens.”*

P12, Male patient, community-based care

#### Livelihoods

Illness and admission to hospital often had catastrophic effects on peoples’ income and financial stability. Increased household costs were primarily for food and transport, but overall these costs were much lower for those people who were discharged from hospital:

*“If someone has been admitted to hospital, transport costs can make them poor because it means they have two households: they should take care of home and come here to see the patient.”*

P29, Female nurse

The impact of greater financial demands during admission was compounded by not being able to work, and this was particularly evident for patients and guardians who remained in hospital for the full two months of treatment. Experiences were affected by prior employment status. Those who had fixed employment had variable experiences during illness – many were not paid, some lost their jobs entirely, and others managed to continue going to work. Those running small informal enterprises sometimes lost their livelihood, but for others the increased flexibility meant that they were able to make arrangements to continue the business. This was also dependent on social networks, which, if strong, could be called upon to assist, but if weak meant no help. Many of those receiving community-based care were able to return to work, and in some cases guardians even went to the patients work place in order to deliver injections.

Coping mechanisms for financial difficulties most often involved borrowing money, but trial participants also described having to sell items that they owned. Borrowing money was always related to the costs associated with hospital admission:

*“I was full of worries, saying to myself, ‘I obtained a loan recently and they are saying I should be admitted.’ My life was sad all the time thinking about the loan and the sickness”*

P24, female patient, hospital-based care

Rather than taking formal loans, once again people relied heavily on their existing social support systems. Both men and women, young and old borrowed frequently and there were no clear patterns differentiating groups in terms of financial coping. Financial concerns were responsible for a large amount of the emotional distress suffered during treatment, especially for those who remained in hospital for prolonged periods.

### Trust

#### Interpersonal relationships

In general, relationships between patients and guardians were strong and very positive in both the hospital and community groups. The job of the guardian included practical activities such as washing, bathing and feeding; helping with medical care for example giving tablets (or injections), and monitoring patients’ condition; as well as providing emotional support and companionship. Such close interactions often strengthened the relationship between the two. This was particularly the case if the guardian was injecting as many patients felt particular gratitude for the job their guardians were doing. However, the sacrifices made by guardians in both groups were often enormous:

*“The 60 days hurt her more than me because it reached a point of her lying to me that she had eaten already just to ensure that I ate the little food that became available when actually she had not eaten. She would stay without eating.”*

P7, Male patient, hospital-based care

Although a few guardians felt pressure from the patient or other family members to take on the role, the vast majority felt some duty to care for the sick person which was instinctive. Occasionally, guardians were motivated by the fear that the community would think badly of them or see them as ill-wishers to the sick; or the hope that their good deed would be reciprocated in the future.

*“It denotes love, the love that you have for that person, because it’s hard to help somebody you don’t love. But if you love that person and help him wholeheartedly, I think your relationship with that person improves. And that person accepts you because he knows you are going to help him.”*

*P14, Male guardian, community-based care*

Whilst age played some role in choice of guardian, with older people being less confident to learn how to do injections, the data do not suggest that gender did - men and women both took on the role frequently and just as willingly. Not only performing injections (which may have been seen as having higher status), but taking care of the daily needs of the patient such as washing and dressing those in hospital, seemed to be entirely compatible with masculinity.

In general, families pulled together to support both guardian and patient in hospital. However, family dynamics often suffered for patients in hospital, primarily as many felt that they were neglecting their children during the long admission, and this caused sadness and fear.

*“I had a small kid … the time I was in the hospital the kid looked like a child with no parents. She would go and wait for food in people’s homes. She would move around uncontrollably … it would bother me greatly.”*

P16, Female patient, hospital-based care

On the other hand, strong relationships developed between those living on the ward, where a short-term community of patients and guardians was seen to develop. As many of the patients were in hospital merely to receive injections, some were well enough to take on guardian roles for other patients:

*“He did not have a guardian, and once I bathed him. And the example I demonstrated influenced all the other people around to follow suit. When it was morning, one would go and warm some water for him … we kept doing that until things started improving for him.”*

P7, Male patient, hospital-based care

#### Community interactions

Participants relied heavily on the support of their communities to look after children left alone at home, visit those in hospital, and even provide financial help. The burden of the responsibilities placed on friends and neighbours was significantly reduced when patients were managed at home, which was perceived by those in the community group as very positive. Frequently, but not always, patients felt well enough to care for their children once discharged; but there was also a major impact for guardians who were able to care for their patient and children together at home. Again, pre-existing roles within communities influenced the support people received - for those who had weaker social networks on which they could not rely, more formal networks were sometimes able to step in, for example religious groups or work- based communities.

Issues around disclosure of health status and stigma featured, but were related more to HIV than to TB. Admission to the TB ward for a prolonged period was sometimes effectively seen as a disclosure of HIV status. In one case a participant claimed that “if you have been admitted to hospital for two months people know that you are also HIV positive.” There were concerns, mainly from health care workers rather than trial participants, that talk in neighbourhoods about guardians doing injections would be stigmatising. Once again, this abstract perception was not borne out in concrete experience - in reality, guardians in the community group felt that they earned respect from the community for the helping role they were playing.

#### Beliefs about healthcare

Trial participants described mixed experiences of their interactions with healthcare workers. A small number complained of negative interactions with nurses, for example guardians feeling that their patients were being neglected (e.g. delays in changing a drip); and being shouted at for complaining or asking for help. However, it was well recognised by both patients and guardians as well as the healthcare workers themselves that the overcrowded environment on the ward made the job of delivering nursing care hard.

It was believed that the delivery of injections was the responsibility of doctors and nurses, but most recognised that guardians could take on the job, because they saw advantages to home-based care. However, it was clearly felt by trial participants and key informants that one advantage of hospital-based over community-based management was being able to access help quickly if the patient’s condition deteriorated. Once again, this theoretical concern was not realised in any actual experience, as no trial participants described a situation in which they were, or even felt as if they were, stuck in the community with no access to care.

There were no instances where guardians administered injections to people other than the patient they were trained to inject, and all said that they would not consider doing so without instruction from the hospital. Not only did all guardians trained to do injections deny injecting members of their communities, they were able to give very clear reasons why they would never do so, unless instructed by a healthcare worker:

*“No. I was trained to inject the patient for who I was a guardian. You are responsible only for your patient... you can’t do that on your own, it would be an offense for you are not sure about the medications required for that person... I also learnt that if you are to inject somebody, you need to know how much they are weighing and their weight determines their dosage.”*

P23, Male guardian, hospital-based care

Overall, experiences of community-based care during the intensive phase of TB-retreatment were overwhelmingly more positive than those of hospital-based care:

*“That part [performing injections] needs to be fulfilled by the nurses because it is their job. But a guardian does this job because people can’t stay in the hospital for 2 months … it is a place where you end up getting other infections instead of getting well … you find that most of your relatives are not able to go there because of financial constraints … they saw it was possible for a guardian to go and inject a patient at home if properly trained. They saw it was a good idea.”*

P15, Female guardian, hospital-based care

## Discussion

Evaluation of the lived experiences of patients and their guardians during the injections for TB retreatment produced three main categories of description: medical issues, the physical environment and trust. Experiences were often influenced by context of the patients’ and guardians’ prior roles within their communities and households, and resulted in a range of emotional responses such as sadness, fear, anger, happiness and acceptance. Community-based management affected experiences in all three categories of description, and again these experiences were impacted by previous roles and backgrounds.

A striking finding is the disastrous effect that admission to hospital has on peoples’ ability to continue normal daily life, and in particular the catastrophic consequences of this on their livelihoods. Once discharged, those in the community were relieved of many of their expenses and more easily able to return to their usual routines, thus relieving some of the economic pressures and associated emotional stress. Reducing catastrophic costs associated with TB is a key element of the post 2015 Global TB strategy [9], as catastrophic costs push households further into poverty, which is associated with the development of TB [10], delayed diagnosis [11], poor adherence and worse clinical outcomes [12].

Socially, the advantages of a community-based model were also evident. Patients, guardians, family members and local communities were all affected by having to look after those in hospital. Social capital is widely recognised to be a crucial component of peoples’ ability to access care, engage with health systems and remain in treatment. Although solidarity from social networks or ‘the clanship system’ [13] have traditionally been seen as fundamental to the structure of many African societies, it has also been reported that the main source of assistance (and therefore duty) lies within the household rather than with the wider community [14]. In line with this, those with strong social networks admitted to hospital in this study were able to rely on both families and broader social groups to help with the practicalities of being sick, whereas those with weaker social networks suffered. ‘Social networks’ were most often informal, but also included networks based around a workplace or religious community.

The commitment and sacrifice shown by guardians was often enormous. The motivations for people to volunteer as community health workers have previously been well described and include both financial and non-financial reward [15, 16]. However, the factors that influence how a family member or friend volunteers to take on a guardian role, are less well understood, and this study goes some way to describing those factors.

Reciprocity is a key concept defining social interactions. It includes motivation for looking after the sick, and is particularly relevant in Africa [17]. The importance of reciprocity is once again demonstrated by this study in multiple situations, including the motivation for taking on the role of guardian in hospital and at home; community responsibility for the sick; and patients looking after other unwell people on the ward.

In contrast to previous studies [18, 19], the role of guardian was not particularly gendered with men and women taking equal responsibility for all aspects of care. It may have been that the task of administering injections was seen as more important (and therefore more appropriate for men to take on) than other traditional roles played by guardians such as washing and cleaning. However, by and large, male guardians enrolled into the trial continued in their guardian roles even after being randomised to hospital-based care.

There were a few instances where people encountered discrimination, but this was not a central theme and the examples were all related to fears around TB being associated with HIV, which have been well illustrated in previous studies [20–22]. It was acknowledged that admission to hospital often disclosed HIV as well as TB status, particularly if the admission was long. The research was conducted in two urban areas with high burdens of HIV and TB, therefore the understanding and assumptions which people were able to make may apply less in settings where the prevalence of HIV is lower. Importantly, there was only perceived, but not realised, stigma around guardians delivering injections in the community; and no concerns were expressed about transmission of TB from patients receiving injections at home.

Emotionally, there was a broad range of experiences, which arose from medical, practical and social factors. Experiences of people on the ward were often distressing, with issues around overcrowding, lack of space, and hygiene being paramount. Other common feelings were the sadness and anxiety around financial issues and the neglect of children at home, which is consistent with previous literature [23].

The concept of ‘the sick role’ acknowledges illness as a social and cultural phenomenon, in which the sick person adheres to a specific role within society, which brings with it certain rights and responsibilities. Data from this study clearly demonstrate patients taking on a sick role, as the nature of their position in society and relationships with others are completely changed by their illness. In this study, it is clear that patients in the community feel able to break free from the sick role whereas patients in hospital maintain the role. Moreover, one of the aspects of community-based care which contributed most to patients’ emotional well-being (and, according to some, physical well-being) was the ability to practically and psychologically move away from this role.

Experience of medical care varied. Participants described well known barriers and facilitators to accessing care such as previous experience of TB [24], costs [25], accessing informal providers [26], stigma [27], and HIV [28].

For both hospital and community-based care, trust in the health system is crucial. The importance of trust in the health system is that it increases a patient’s willingness to seek care, enhances ability to engage with treatment, improves relationships between patient and clinician, facilitates disclosure, and may grant patients more autonomy in decision-making [29]. Theories of trust can be framed around both trust in the individual and in the system. In the current study we see that indeed trust in the healthcare system was primarily determined by interactions with healthcare workers, and that failure of these representatives to earn the respect of trial participants, impaired the relationship between the two. Conversely, the feeling of security generated by being in hospital represented the trust that people had in the ability of clinicians and nurses to provide help if a patient was unwell.

One of the major advantages of community-based care was the opportunity to escape the overcrowded and often unclean conditions on the ward. The distress that this caused to patients and guardians is in keeping with previous literature in which patients with MDR-TB have expressed a preference for home-based care, citing transmission of infection and lack of psychosocial support as problems with hospital-based care [30].

### Study limitations

The study had some limitations. Data were collected almost exclusively at a single time point, just after completion of injections. It may have been that experiences changed during the period of the intervention and also that both community-based management and hospital-based management may have longer term effects on participants beyond the completion of injections. Additionally, although every attempt was made to sample participants who experienced adverse events during the trial, only two participants (one patient and his guardian) were interviewed who had complications whilst in the community. The impact of urban or rural residence was not specifically examined in this study, with only three of the interviewees from rural areas.

### Stakeholder concerns addressed and implications for policy

There had been a number of concerns raised by those consulted at the start of the TB-RROC trial, which this evaluation have helped address. Firstly, there was a fear that guardians simply would not deliver the injections at home. Not only did trial participants report excellent adherence, this was made more convincing by the (often in-depth) explanations they were able to provide as to why they would not miss doses.

Secondly, there had been a worry that guardians would not be competent to deliver injections. Although many were also initially apprehensive, all of those who took part were able to overcome these concerns and embrace the responsibility.

Thirdly, the issue of access to medical care during treatment at home had been raised. Very few participants described needing to access care whilst in the community but those who did were able to contact the study team, and in the one situation where admission was necessary, this was easily arranged and the ultimate outcome was positive.

Finally, it had been suggested that training people to do injections might create a cadre within society of ‘lay injectors’ who would be expected to provide injections for people within their communities. The data from this study strongly suggest that the risk of that happening would be very low.

These data were used to provide the Malawi National TB Programme with additional information which could be used to modify and improve the model of community-based care for patients requiring injections as part of TB treatment. Recommendations included: developing more concrete clinical guidelines for selecting patients to be managed in the community; setting out suggestions for choosing guardians to do injections; and ensuring clear pathways to access care from the community if patients become unwell.

## Conclusion

Overall, these data demonstrate that the experience of community-based management offers many advantages over hospital-based management. Concerns raised by participants as well as stakeholders have been both recognised and addressed. Although there were abstract worries about issues in the community such as adherence, relationships between patients and guardians and the ability of lay people to deliver injections safely, none of these were borne out in real experiences. Community-based management offers a number of benefits, including fewer financial difficulties, improved emotional well-being, and the ability to continue social interactions and daily activities.

### Additional file

Additional file 1: TB-RROC topic guides. (DOCX 101 kb)

## Figures and Tables

**Fig. 2:**
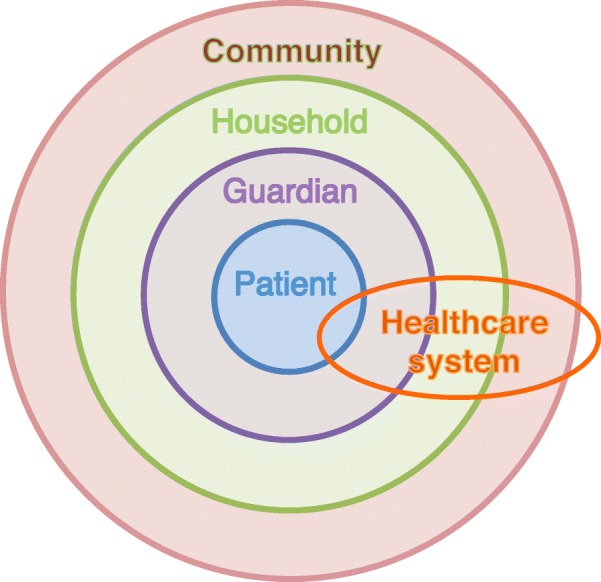
Theoretical framework

## Appendix 2

**Table 2:** Sampling matrix

Patients				Guardians			
20				20			
Hospital-based care		Community-based care		Hospital-based care		Community-based care	
10		10		10		10	
Male	Female	Male	Female	Male	Female	Male	Female
5	5	5	5	5	5	5	5

## Abbreviations

HIV: Human immunodeficiency virus
TB: Tuberculosis
TB-RROC: TB Retreatment Regimen Outcomes and Care Study
