# Supplementary material for: A qualitative evaluation of hospital versus community-based management of patients on injectable treatments for tuberculosis
Source: BMC Public Health. 2018 Sep 17;18:1127. doi: 10.1186/s12889-018-6015-3 (PMC6142700; doi:10.1186/s12889-018-6015-3)
Supplement: Supplementary file 1 — TB-RROC topic guides. (DOCX 101 kb) [file 12889_2018_6015_MOESM1_ESM.docx]

**Supplementary material**

**TB-RROC Qualitative Study: Topic guide for Patients**

Thank you for agreeing to talk to me. I am interested in learning about what it’s been like getting treatment for TB. I will be recording the interview, just so that I can make sure I have an accurate record of our conversation. If you feel uncomfortable at any time we can stop, and if we are talking about something you don’t want to talk about just tell me and we can skip it.

- What does the participant know about TB
- What are the participant’s experiences of TB treatment
  - Describe your treatment to me.
  - Tell me about your injections.
  - What is it like being in hospital.
  - What is it like getting treatment at home.
  - What is it like being in your community when you are taking TB treatment.
  - How do you feel about having TB treatment.
  - Did you feel differently about TB at the beginning of your treatment compared to now. Tell me more about that.
- I would like to hear more about the healthcare workers you met during treatment. Can you tell me about them
  - How often did you have contact with healthcare workers.
  - What role did they play in your treatment.
- How, if at all, has having TB treatment affected your life.
  - Work
  - Relationships – spouse, children, friends
  - Financial situation
- What things influence people to continue taking TB treatment.
  - What things help somebody to take TB treatment.
  - What things make it difficult for somebody to take TB treatment.
  - What things would make it easier for you to take TB treatment.
- Do you talk to anyone about your TB treatment.
  - Who do you talk to.
  - Tell me what it’s like talking to other people about TB
- What do you think will happen to your health in the future.
- Those are the main topics I had planned to cover, but is there anything else you would like to tell me about? Is there something we have missed that you think I should know?

**TB-RROC Qualitative Study: Topic Guide for Key Informant Interviews**

- Role of the Key Informant in TB care
- Views of inpatient care for TB patients
  - Possible problems & advantages for patients
  - Possible problems & advantages for guardians/friends/relatives
  - Possible problems & advantages for Health care systems
- Opinions about the intervention
  - Effects for patients
  - Effects for guardians/friends/relatives
  - Effects for communities
  - Effects for health care systems
- General view of the intervention

**TB-RROC Qualitative Study: Topic Guide for Observations in Hospital**

- Setting and environment of the hospital – male & female wards, nursing station, TB office
- General condition of the patients – physical and psychological
- Behaviours of patients on the ward
- Relationships between patients – interactions, discussions
- Behaviours of guardians – on and around the ward
- Interactions between patients and guardians
- Relationships between patients, guardians and healthcare workers
- The process of injection administration on the ward

**TB-RROC Qualitative Study: Topic Guide for Observations in the Community**

- Setting and environment of the household
- General condition of the patient – physical and psychological
- Interaction between patient and guardian
- Interaction between patient and the rest of the household/community
- Interaction between guardian and the rest of the household/community
- The process of the guardian administering the injection
- Reaction of the patient, guardian, household members, wider community to the fieldworker
